# A trivalent gastric cancer antigen conjugated to a diphtheria toxin epitope synergizes with an NKT cell agonist to boost antitumor immunity

**DOI:** 10.3389/fimmu.2026.1903458

**Published:** 2026-09-24

**Authors:** Jing-Jing Du, Rong-Ling Liu, Yao-Wei Bai, Jin-Xuan Su, Yuan-Mei Zheng, Zhen-Hong Su, Shi-Hao Zhou, Jun Guo, Dong-Yuan Wang, Ru-Yan Zhang

**Affiliations:** 1 Hubei Provincial Key Laboratory of Occurrence and Intervention of Kidney Diseases, Hubei Provincial Engineering Research Center of Immunotherapy Drugs for Renal Tumors, Hubei Polytechnic University, College of Medicine, Huangshi, China; 2 Department of Pharmacy, Union Hospital, Tongji Medical College, Huazhong University of Science and Technology, Wuhan, China; 3 National Key Laboratory of Green Pesticide, International Joint Research Center for Intelligent Biosensing Technology and Health, College of Chemistry, Central China Normal University, Wuhan, China

**Keywords:** diphtheria toxoid, gastric cancer antigen, NKT cell agonist, peptide vaccine, tumor immunotherapy

## Abstract

The gastric cancer-associated antigen MG7-Ag is aberrantly expressed in multiple gastrointestinal malignancies and represents a promising broad-spectrum target for pan-gastrointestinal tumor peptide vaccines; however, its clinical application is hampered by low immunogenicity. To overcome this limitation, we covalently conjugated a diphtheria toxin-derived T-helper epitope to the trivalent antigen MG3, which was constructed by tandem repetition of the monovalent MG1 peptide from the MG7-Ag. A liposomal vaccine was subsequently developed by incorporating DT-MG3 together with the natural killer T (NKT) cell agonist α-galactosylceramide (αGalCer). Immunological evaluation demonstrated that DT-MG3 plus αGalCer elicited significantly higher specific IgG titers than DT-MG3 alone or the unconjugated MG3/αGalCer combination. The vaccine also promoted IFN-γ/IL-4 secretion and boosted CD4^+^/CD8^+^ T cell responses to elevate antigen immunogenicity. Moreover, the high-titer specific antibodies induced by the vaccine also efficiently bound to EAC tumor cells and induced complement-dependent cytotoxicity. In the EAC ascites model, vaccination with DT-MG3/αGalCer substantially reduced malignant ascites accumulation and prolonged the survival of tumor-bearing mice. Collectively, this novel anti-gastric cancer vaccine based on the covalent conjugation of a diphtheria toxin epitope to the trivalent MG3 antigen, provides valuable insights for the rational design of potent and safe fully synthetic peptide-based anti-tumor vaccines.

## 1 Introduction

Gastric cancer (GC) is one of the most prevalent malignant tumors of the digestive system, ranking fifth in incidence worldwide and carrying a high mortality rate, thus representing a severe threat to human health (1, 2). Although a comprehensive therapeutic regimen centered on surgical resection combined with chemotherapy, targeted therapy, and immunotherapy has been established, patients with advanced gastric cancer still face limited effective interventions and an extremely poor prognosis (3). Therefore, novel therapeutic strategies with improved targeting efficacy and biosafety are urgently needed. Tumor vaccines have been recognized as a key direction for the precision treatment of advanced gastric cancer, as they aim to induce durable immunological memory that can sustain antitumour immune responses beyond the period of active treatment, potentially providing prolonged protection against tumour recurrence (4–6).

However, the development of highly effective GC vaccines has been hindered by the shortage of ideal tumor-associated antigens. Monoclonal gastric cancer 7 antigen (MG7-Ag), a GC-associated tumor antigen, is highly expressed in gastric cancer tissues (7, 8). Furthermore, its expression is closely associated with tumor invasion, metastasis, and prognosis; therefore, it represents a promising target for vaccine design (9, 10). In addition, aberrant expression of MG7-Ag has been observed in various other gastrointestinal malignancies, including colorectal cancer and esophageal cancer, suggesting that MG7-Ag represents a promising broad-spectrum antigen for the development of pan-gastrointestinal tumor peptide vaccines (11).

Various immunotherapeutic strategies based on MG7-Ag have been developed (12, 13). Nevertheless, the weak immunogenicity of MG7-Ag remains a major limitation for GC vaccine development. Specifically, monovalent MG7-Ag peptide-based vaccines (defined as MG1) exhibit limited activation of cellular immunity, which is essential for antitumor effects (13–16). Moreover, the antigen-presentation efficiency of native MG7-Ag epitopes is relatively low; they are inefficiently recognized and presented to T cells by dendritic cells (DCs), thereby impeding the initiation of effective immune responses (17). Consequently, monovalent MG1 vaccines alone are insufficient to activate robust systemic antitumor immunity. In contrast, previous studies have demonstrated that multivalent vaccines elicit markedly stronger antitumor effects than monovalent vaccines and effectively inhibit tumor growth in mouse models (11, 13). Therefore, enhancing antigen clustering through covalent tandem repeats of antigenic peptides, defined as the spatial organization of multiple epitopes to facilitate B-cell receptor cross-linking and enhanced signalling, is required to achieve optimal immune efficacy in GC vaccines (11).

Fully synthetic vaccines constructed with protein-derived T-cell epitope peptides exhibit high biosafety, efficiently activate T cells, and avoid excessive immunogenicity (18, 19). Diphtheria toxoid (DT) and its non-toxic cross-reactive material CRM197 are clinically approved carrier proteins that have been widely used in licensed conjugate vaccines against pathogens (20). The T-helper epitopes derived from these carrier proteins can be exploited in peptide vaccine design to provide CD4^+^ T-cell help, which is important for the activation and differentiation of antigen-specific B cells and CD8^+^ T cells. Activated CD4^+^ T cells provide critical help to DCs through CD40L-CD40 interactions, a process that licenses DCs for efficient cross-presentation and priming of CD8^+^ T cells (21, 22). The DT-derived T-helper epitope, when presented to naïve CD4^+^ T cells via the MHC class II-restricted pathway, induces Th1 differentiation and upregulates the expression of multiple antitumor cytokines, including interferon-γ (IFN-γ), thereby potentiating cytotoxic T lymphocyte-mediated cellular immunity (23). Previous studies have demonstrated that DT epitope conjugation markedly enhances the antitumor activity of peptide vaccines against tumor, providing a rational and feasible strategy for developing vaccines that covalently link a helper T-cell epitope to gastric cancer antigens (19).

Adjuvants are regarded as critical components of antitumor peptide vaccines, and their immunogenicity-enhancing effects are achieved by activating innate immune cells, modulating the immune microenvironment, and improving antigen-presentation efficiency (24–27). α-Galactosylceramide (αGalCer, also known as KRN7000) is a synthetic glycolipid agonist of natural killer T (NKT) cells, developed as a synthetic analogue of agelasphins derived from the marine sponge *Agelas mauritianus* (28–31). It is presented to NKT cells in a CD1d-restricted manner, and rapid activation of NKT cells is induced, accompanied by the secretion of various cytokines such as IL-4 and IFN-γ (32). The rapid kinetics of NKT cell activation, producing cytokines within minutes to hours, contrasts with the slower responses of conventional CD4+ T cells. This temporal advantage may play an important role in effectively bridging innate and adaptive immunity. Additionally, CD40L expression on activated NKT cells contributes to DC licensing, further enhancing antigen cross-presentation and CD8+ T-cell priming. In addition, NKT cells facilitate B-cell proliferation, antibody class switching, and affinity maturation, serving as a critical bridge between innate and adaptive immunity (33, 34). Previous studies have validated that αGalCer, used as an adjuvant in combination with tumor-associated antigens, can substantially enhance the antitumor efficacy of vaccines in melanoma, lung cancer, and GC models and induce durable antigen-specific immune memory (35–38).

Based on these advancements, a gastric cancer vaccine was constructed by covalently conjugating the DT-derived the DT-derived T-helper epitope DT_331-345_ (sequence: QSIALSSLMVAQAIP) (19), to the trivalent MG3 antigen (KPHVHTKPHVHTKPHVHTK), which consists of three tandem repeats of the monovalent MG1 peptide (KPHVHTK) (11, 16). Multivalent antigen display facilitates spatial clustering of epitopes, which qualitatively enhances B-cell receptor cross-linking and signalling as compared with simply increasing the epitope dose in a monovalent format. In addition, the covalent linkage between the DT helper epitope and the MG3 antigen promotes co-delivery and presentation of both components by the same antigen-presenting cell, potentially facilitating more efficient linked help for T-B collaboration than simple physical mixing of the two components. The NKT cell agonist αGalCer was incorporated as an adjuvant. This design strategically combines covalent conjugation of the DT epitope with co-delivery of the NKT cell agonist, aiming to achieve a potent synergistic effect that enhances both the immunogenicity and the antitumor efficacy of the vaccine (**Figure 1**). DT-MG3, as an amphiphilic peptide conjugate antigen, can co-assemble with the amphiphilic αGalCer adjuvant, the auxiliary lipid DSPC, and cholesterol to form liposomes. This co-encapsulation allows both the antigen and the adjuvant to be delivered to the same antigen-presenting cells, enabling the co-delivery required for coordinated activation of both innate and adaptive immune pathways. This study was designed to elucidate the mechanism by which the DT epitope synergizes with the αGalCer adjuvant to boost the immunogenicity of the MG1 antigen-based vaccine, thereby providing experimental evidence and a novel strategy for the development of targeted peptide-based antitumor vaccines against gastric cancer.

**Figure 1:**
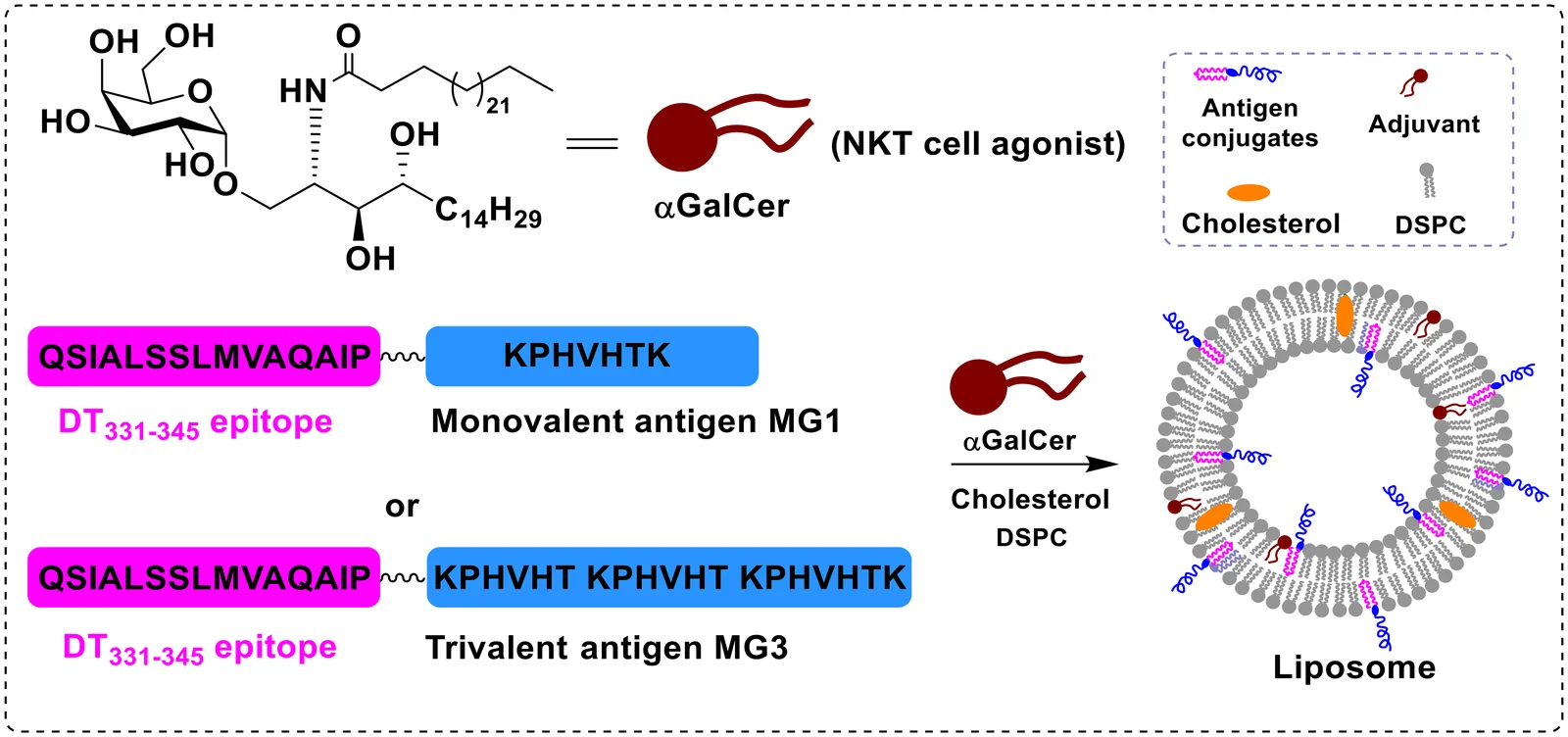
Schematic illustration of the gastric cancer vaccine constructed by conjugating the DT-derived helper T-cell epitope to the peptide antigens and adjuvanted with αGalCer.

## 2 Materials and Methods

### 2.1 Synthesis of the antigen

All peptides were synthesized via solid-phase peptide synthesis (SPPS) using the Fmoc-protection strategy (**Scheme 1 A, B**). The B-cell epitope of the monomeric gastric cancer antigen MG1 (MG7-Ag) with the sequence KPHVHTK (16), was covalently conjugated with diphtheria toxin-derived DT_331–345_ epitope peptide (QSIALSSLMVAQAIP) to construct the DT-MG1 conjugate. The trivalent antigen MG3 ((KPHVHTKPHVHTKPHVHTK)) was also constructed by multimerization of MG1 monomers and further conjugated with DT_331–345_ peptide to yield DT-MG3. Amino acid coupling was performed in anhydrous DMF, using PyBOP and DIPEA as coupling reagents. Cleavage was performed with 95% TFA/2.5% TIS/2.5% H_2_O for 2 h. Crude peptides were obtained by filtration, concentration, and ether precipitation, and then dissolved in acetonitrile/water for purity analysis. Purification was performed by reversed-phase high-performance liquid chromatography (RP-HPLC), and the DT-MG1 and DT-MG3 peptides were isolated with yields of 32% and 25%, respectively. The classical NKT-cell agonist αGalCer was synthesized using the well-established protocol previously developed in our group (31, 39, 40).

**Scheme 1:**
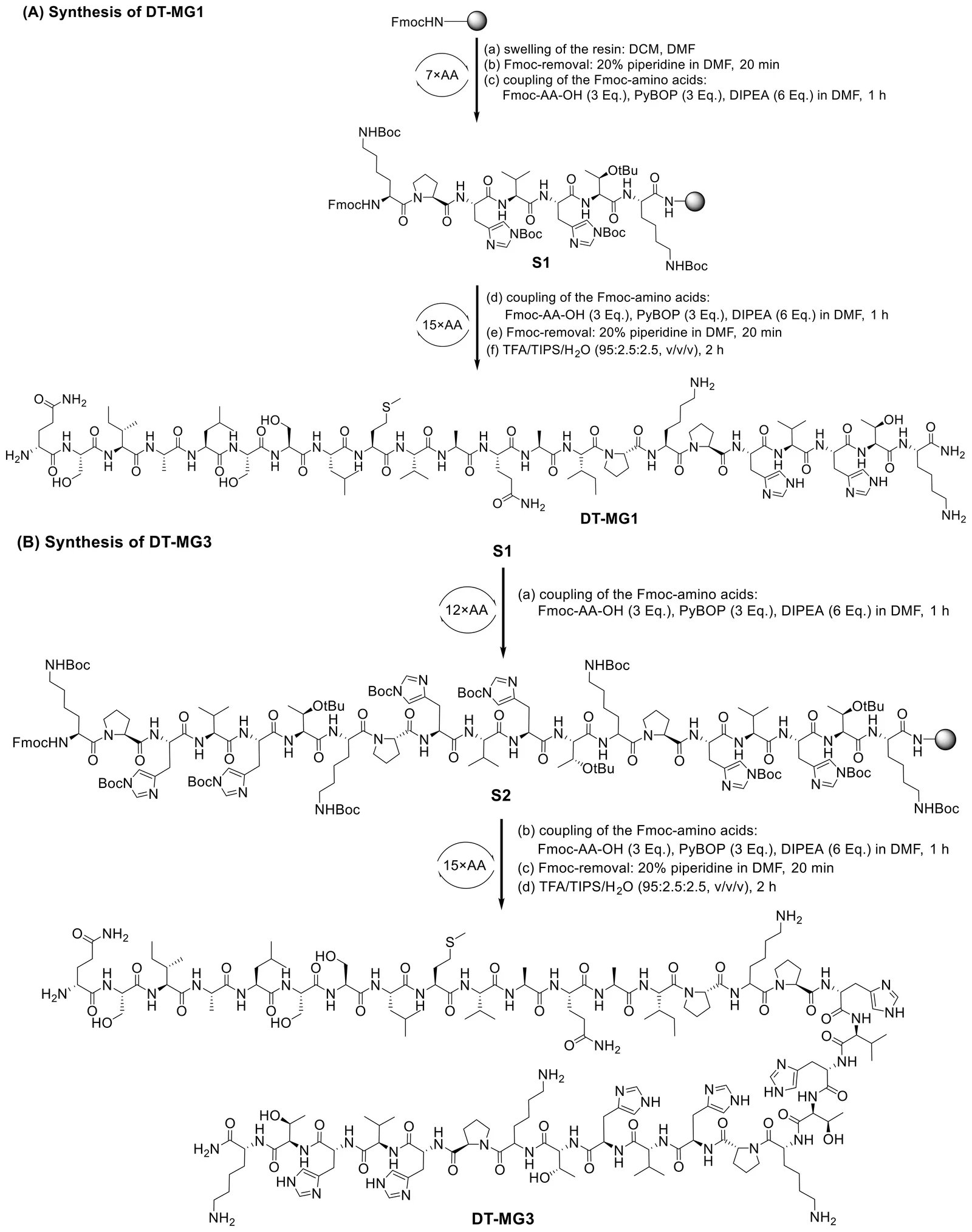
Preparation of the peptide antigens: **(A)** DT-MG1; **(B)** DT-MG3.

### 2.2 Vaccine preparation

Anti-gastric cancer vaccines were prepared using the thin-film dispersion method, with the molar ratio of antigen/adjuvant/distearoylphosphatidylcholine (DSPC)/cholesterol fixed at 10:5:50:40. The dosage of the adjuvant αGalCer was strictly controlled at 2.3 nmol (1.97 μg), which prior studies have established as the optimal effective dose for this adjuvant, as excessive αGalCer may lead to NKT cell anergy, potentially compromising immune system activation and thereby reducing the overall immunogenicity of the vaccine (31, 40). Seven different formulations were prepared: PBS alone as the blank control, groups containing a single antigen (DT-MG3 or DT-MG1), and mixture groups (MG3+αGalCer, DT-MG3+αGalCer, DT-MG1+αGalCer, and MG1+αGalCer). For each formulation, DSPC (23 nmol), cholesterol (18.4 nmol), αGalCer (2.3 nmol), and the respective antigen (DT-MG1, DT-MG3, MG1, or MG3, each at 4.6 nmol) were dissolved in a suitable volume of dichloromethane/methanol (DCM/MeOH, 1:1, v/v). Following brief sonication, the organic solvents were completely removed by rotary evaporation under reduced pressure, yielding a uniform lipid film on the vial wall. An appropriate volume of HEPES buffer was then added, and the mixture was vigorously vortexed for 2 min to ensure thorough dispersion, followed by sonication for 15 min in an ultrasonic cleaner (100 W, 40 kHz) to enhance uniformity.

### 2.3 Mice immunization

SPF female BALB/c mice (4–6 weeks old) (laboratory animal license No.: SCXK(E) 2025-0018) were randomly assigned to experimental and control groups. Mice (n = 5 per group) received intraperitoneal injections of the candidate vaccines on days 1, 15, and 29. Serum samples were collected from the mice on days 14, 28, and 42 for antibody titer and subtype analysis. In addition, mouse splenocytes were collected on days 42 and isolated for subsequent intracellular cytokine staining (ICS) and cytotoxicity assays. For the ascites tumor model experiment, mice were acclimatized for one week and then randomly divided into four groups. Tumor models were established by intraperitoneal injection of 100 µL of logarithmic-phase EAC (Ehrlich ascites carcinoma) ascites cells resuspended in PBS. From day 8 after model establishment, treatments were administered intraperitoneally every other day for a total of four injections. All animal experiments were conducted in strict accordance with laboratory animal welfare and ethical guidelines. At the end of the experimental period, mice were humanely euthanized by cervical dislocation performed by trained personnel.

### 2.4 Cytokine level detection

The experimental group was intraperitoneally immunized with 200 μL candidate vaccine on days 1, 15, and 29, while the control group received equal volume of PBS (total three immunizations). Serum was isolated from the mice and collected 2 h post-primary immunization and stored at -20 °C. ELISA was used to detect serum IFN-γ and IL-4 levels, 96-well plates were coated with IFN-γ or IL-4 capture antibodies, blocked, incubated with serially diluted serum and cytokine standards,. followed by detection antibodies, streptavidin-HRP and TMB substrate. The reaction was terminated with 2 M H_2_SO_4_ and absorbance at 450 nm was measured to calculate cytokine concentrations.

### 2.5 ELISA for evaluating antibody levels

Female BALB/c mice (4–6 weeks old) were randomly divided into experimental and negative control groups. The experimental group was intraperitoneally immunized with 200 μL candidate vaccine on days 1, 15, and 29, while the control group received an equal volume of PBS. Serum was collected on days 14, 28, and 42, separated by centrifugation, and stored at -20 °C. For ELISA, 96-well plates were coated with D-biotin-Avidin (MG1 0.1 mg/mL) at 4 °C overnight, blocked with 1% casein, incubated with serially diluted sera, then HRP-conjugated anti-mouse IgG, IgM, IgG1, IgG2a, IgG2b, IgG3. After washing, TMB substrate was added for 5 min in darkness, and the reaction was stopped with 2 M H_2_SO_4_. OD450 was measured to quantify antibody titers.

### 2.6 Flow cytometry analysis

Logarithmic-phase EAC cells were harvested, washed with PBS, and digested with 0.25% trypsin-EDTA at 37 °C. The cell concentration was adjusted to 1 × 10^6^ cells/mL. After centrifugation, the cells were resuspended in PBS containing 1% FBS, incubated with mouse serum (diluted 1:50) on ice for 1 hour, and then stained with Alexa Fluor^®^ 488-conjugated goat anti-mouse IgG (H+L) (diluted 1:50 in FACS buffer) in the dark for 30–40 minutes. After washing and filtration, fluorescence intensity was measured by flow cytometry to evaluate the binding efficiency of the antiserum.

### 2.7 CDC assay

Logarithmic-phase EAC cells were seeded into 96-well plates at 8,000 cells/well and incubated overnight. After washing, mouse serum (1:50 in 1% BSA-PBS) was added at 100 μL/well and incubated at 37 °C for 1 h. After removal of serum and washing with PBS, 100 μL/well rabbit serum (1:5 in 1% BSA-PBS, complement source) was added and incubated at 37 °C under 5% CO_2_ for 2 h. Then 20 μL of 0.5% MTT-PBS was added per well and incubated at 37 °C for 4 h. Supernatant was removed, 150 μL DMSO was added, and shaken for 10 min OD_490_ was measured, and CDC efficiency was evaluated from OD differences between experimental and control groups.

### 2.8 Ex vivo splenocyte cytotoxicity assay

Mice were euthanized on day 42 post-immunization. Spleens were aseptically isolated and homogenized to prepare splenocyte suspensions as effector cells (5 × 10^6^ cells/well). Logarithmic-phase EAC target cells were adjusted to 1 × 10^5^ cells/well. Effector and target cells were mixed in 96-well plates (200 μL/well) and incubated at 37 °C under 5% CO_2_ for 6 hours. Triplicate control wells were included for spontaneous target LDH release, maximum target LDH release, and spontaneous effector LDH release. Lysis buffer was added to the maximum release group 1 hour before termination. After the 12-hour incubation, the plates were centrifuged at 250 × g for 4 minutes; 120 μL of supernatant was then transferred and mixed with 60 μL of LDH reagent. Following incubation at room temperature in the dark, the optical density at 490 nm (OD_490_) was measured. Cytotoxicity was calculated using the following formula: Cytotoxicity (%) = [(Experimental OD - Effector spontaneous OD - Target spontaneous OD)/(Maximum OD - Target spontaneous OD)] × 100%.

### 2.9 Intracellular cytokine staining assay

Mice were euthanized 14 days after the last immunization (on days 42), and spleens were aseptically harvested. After erythrocytes lysis and centrifugation, splenocytes were resuspended in RPMI 1640 medium, and cultured in 24-well plates. Cells were stimulated with MG1 peptide for 2 h, then treated with a protein transport inhibitor and incubated overnight. After washing, cells were stained with anti-CD3/CD4/CD8 antibodies on ice for 30 min, then fixed, permeabilized, and stained with FITC-anti-TNF-α and PE-anti-IFN-γ. Frequencies of TNF-α+ and IFN-γ+ cells in CD3^+^CD4^+^ and CD3^+^CD8^+^ T-cell populations were determined by flow cytometry.

### 2.10 Antitumor immunity assay

BALB/c mice were acclimatized for one week and randomly divided into four groups with five mice in each group: PBS group, DT-MG3+αGalCer group, DT-MG3 single treatment group, and MG3+αGalCer group. An EAC ascites tumor model was established by intraperitoneal injection of 100 μL logarithmic-phase EAC ascites cells resuspended in phosphate buffered saline (PBS). Starting on day 8 after tumor inoculation, intraperitoneal administration was performed every other day for a total of four times. The survival status and body weight of mice were observed and recorded daily to plot the body weight curve. At the experimental endpoint, all mice were euthanized. Dissection and imaging examination were conducted to evaluate the growth of ascites tumor and the degree of organ infiltration. Independent experimental cohorts (n = 5 per group) were set up for survival analysis.

### 2.11 Safety assessment

On day 42 post-immunization, mice were anesthetized, and blood was collected via eyeball extirpation. After clotting at room temperature for 30 to 60 minutes, then centrifuged at 3500 rpm for 10 min, and serum was separated and stored at -20 °C. Serum levels of ALT, UREA and CREA were measured by an automated biochemical analyzer. Major organs were harvested on day 42, fixed in 4% paraformaldehyde for 72 h, embedded in paraffin, sectioned, stained with hematoxylin and eosin (H&E), and examined microscopically.

### 2.12 Statistical analysis

Quantitative data (OD values, cytokine levels, body weight and ascites volume) were compiled and graphically displayed using GraphPad Prism software. Flow cytometry data were analyzed by CytExpert or FlowJo software. Intergroup comparisons were performed by one-way ANOVA with Tukey’s multiple comparison test via GraphPad Prism. Statistical significance was defined as ns (not significant), *P < 0.05, **P < 0.01, ***P < 0.001, ****P < 0.0001. All data are expressed as mean ± standard error of the mean (SEM).

## 3 Results

### 3.1 Characterization of liposome vaccines

The physicochemical properties of the liposomal vaccine formulations were characterized by dynamic light scattering and zeta potential analysis. The results showed that liposomes containing DT-MG3 exhibited an average particle size of approximately 300 nm, whereas those incorporating MG3 without the DT epitope had a smaller average diameter of around 150 nm (**Figure 2A**). Zeta potential measurements indicated that liposomes in all groups possessed a negative surface charge, ranging from –15 mV to –30 mV (**Figure 2B**).

**Figure 2:**
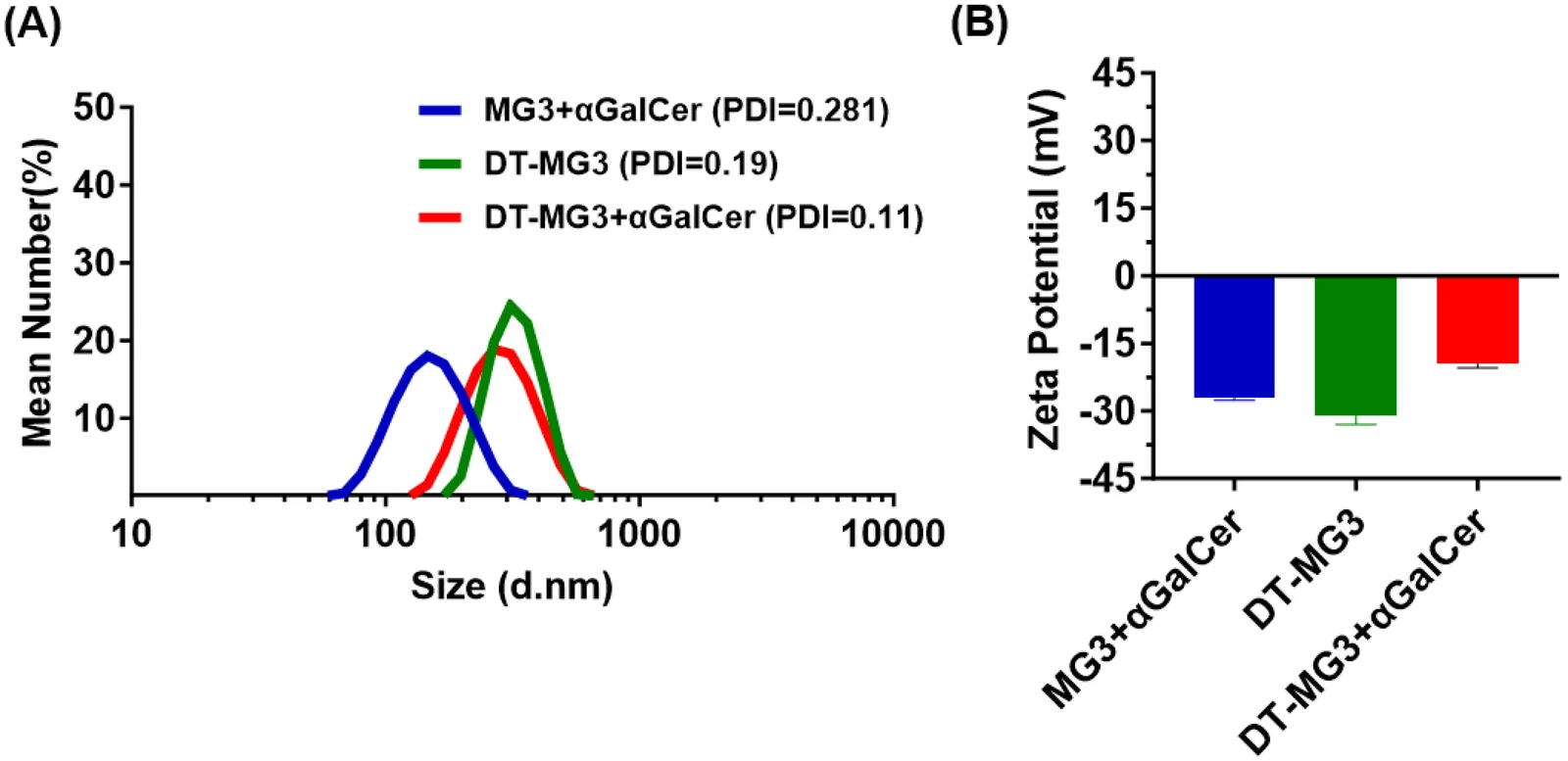
Characterization of particle size **(A)** and zeta potential **(B)** of liposomal vaccine formulations.

### 3.2 Diphtheria toxoid epitope and NKT cell agonist synergistically boost specific immunity

To investigate the polarization status and magnitude of the immune responses induced by the vaccine, the secretion levels of Th2-type cytokine interleukin-4 (IL-4) and Th1-type cytokine interferon-γ (IFN-γ) were evaluated at 2 hours and 24 hours post-primary immunization in mice respectively (41). The experimental results are depicted in **Figures 3B, C**. Experimental results showed that vaccines containing αGalCer promoted the secretion of IL-4 and IFN-γ compared with those without the NKT cell agonist αGalCer, suggesting that αGalCer adjuvant effectively enhances the immunological activity of the vaccine. Among all groups, the DT-MG1+αGalCer vaccine group exhibited the most prominent secretion levels of IL-4 and IFN-γ, with an induction effect more than twice that of the MG1+αGalCer group, which is suggestive of effective iNKT cell activation. These findings demonstrated that the combination of the diphtheria toxin (DT) epitope and NKT cell activation exerted a synergistic effect on the regulation of innate immunity and contributed to high-level secretion of cytokines. Moreover, the liposomal vaccine was suggested to enable the co-delivery of antigens and adjuvants to lymph nodes for synchronized uptake and processing by the same antigen-presenting cell (APC). Given the weak intrinsic immunogenicity and limited immune responses induced by monovalent MG1 antigen, a trivalent MG3 antigen was constructed through tandem repetition of three MG1 sequences (42). Subsequently, the trivalent MG3 antigen was covalently conjugated with the DT epitope and formulated into a liposomal antitumor vaccine with αGalCer served as the adjuvant. This study further explored whether the multivalent antigen strategy could enhance antigen-specific immune responses.

**Figure 3:**
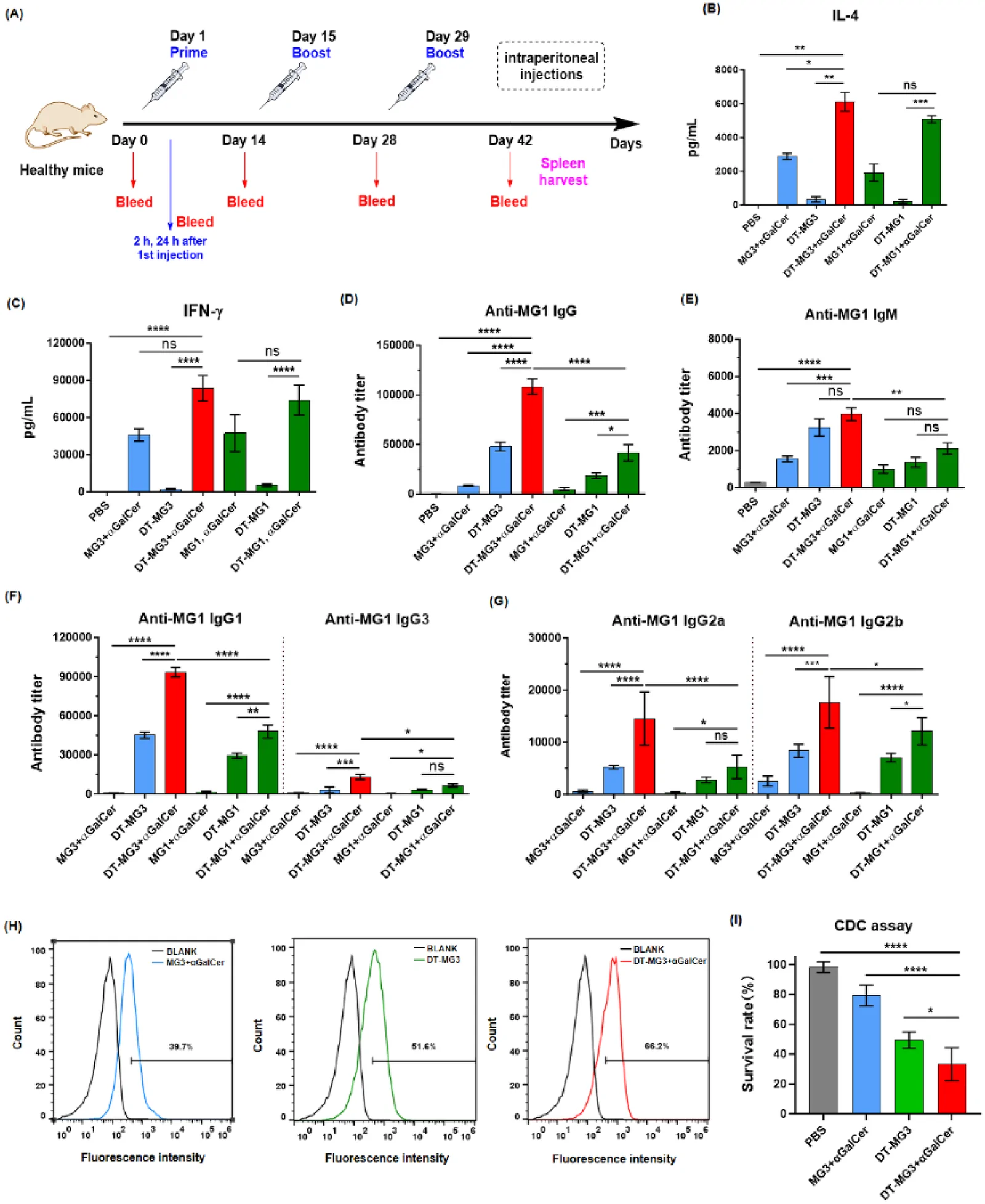
Immunological evaluation of the vaccines. **(A)** Schedule of mouse immunization and blood collection; **(B, C)** Secretion levels of IL-4 (2 h) and IFN-γ (24 h) post-primary immunization; **(D, E)** Anti-MG1 IgG and IgM responses in immunized female BALB/c mice on day 42; **(F, G)** Induction of anti-MG1 IgG subtype antibody titers by vaccines on day 42; **(H)** Flow cytometric analysis of EAC cell recognition by mouse antisera; **(I)** Complement-dependent cytotoxicity against EAC cells determined by MTT assay. Data are presented as mean ± SEM, statistical significance was analyzed by one-way ANOVA with Tukey’s multiple comparisons test: *P < 0.05,**P < 0.01, ***P < 0.001, ****P < 0.0001, and ns indicates no significant difference.

Enzyme-linked immunosorbent assay (ELISA) was used to evaluate the anti-MG1 antibody titer levels induced by vaccines. As shown in **Figure 3D**, the highest IgG titer was induced in mice immunized with DT-MG3+αGalCer, which was 2-fold higher than that of the simple DT-MG3 group and 10-fold higher than that of the MG3+αGalCer group. A similar trend was observed in the DT-MG1+αGalCer group, whose specific IgG titer was markedly higher than those of the single DT-MG1 group and the MG1+αGalCer group. Meanwhile, the anti-MG1 IgG level induced by the DT-MG3+αGalCer group was markedly higher than that of the DT-MG1+αGalCer group, which demonstrated that the multivalent effect of the MG3 antigen in the vaccine significantly enhanced the production of MG1-specific IgG. In addition, low levels of anti-MG1 IgM antibodies were detected in the DT-MG3 and DT-MG1 groups combined with αGalCer, as displayed in **Figure 3E**. This result indicated that T helper cell epitopes exerted a strong immune synergistic effect with NKT cell agonists and effectively strengthened the antigen-specific IgG immune response.

The anti-MG1 antibody titer elicited by the DT-MG3+αGalCer vaccine increased gradually following immunizations (**Supplementary Figure S2**). Compared with day 28, the IgG level in the DT-MG3+αGalCer vaccine group was increased by 2.5-fold on day 42, and was elevated by 5-fold relative to day 14.

To evaluate the immunological characteristics of the various vaccine formulations, IgG subtype analysis was conducted. As shown in **Figures 3F, G**, IgG1 was the dominant subtype of specific antibodies in most groups, including MG3+αGalCer, MG1+αGalCer, DT-MG1, DT-MG3, and DT-MG1+αGalCer. Notably, the DT-MG3+αGalCer group not only increased the level of IgG1, but also promoted the secretion of MG1-specific IgG2a, IgG2b, and IgG3. These results indicated that the combination of an NKT cell agonist with covalent conjugation of the DT epitope effectively enhanced antigen-specific immune responses. Collectively, this vaccine formulation elicited a broad and diverse antibody profile, which may provide more effective immune protection against tumors.

ELISA experiments have demonstrated that the DT-MG3+αGalCer vaccine can induce an effective antigen-specific immune response in mice. To clarify whether the induced antibodies can efficiently recognize and bind EAC tumor cells, EAC tumor cells were co-incubated with serum from immunized mice. Fluorescence intensity was analyzed by flow cytometry, and relevant results are depicted in **Figure 3H**. It was observed that the sera from mice immunized with the three vaccines (DT-MG3+αGalCer; MG3+αGalCer, and DT-MG3) could all effectively recognize and bind to EAC target tumor cells. Among them, the antibody binding rate in the DT-MG3+αGalCer group (66.2%) was significantly higher than that in the DT-MG3 group (51.6%) and the MG3+αGalCer group (39.7%). This result indicated that the T cell epitope derived from diphtheria toxin and the αGalCer adjuvant have a synergistic effect, which can substantially enhance the ability of antibodies to recognize and bind to target tumor cells.

To further investigate whether the induced antibodies could effectively kill EAC tumor cells through complement-dependent cytotoxicity (CDC), the cell viability of tumor cells was evaluated using the MTT assay, the results are presented in **Figure 3I**. The data indicated that the immune serum from the DT-MG3+αGalCer group significantly reduced EAC cell viability, with a cell survival rate of approximately 33%, which was much lower than 49% in the DT-MG3 group and 79% in the MG3+αGalCer group. These findings suggested that the combination of the NKT cell agonist αGalCer and the diphtheria toxin epitope peptide synergistically enhances target cell killing through the complement system.

To further verify whether the antibody recognition of EAC cells was antigen-specific, flow cytometry analysis was performed using B16-F10 cells, a gastric cancer antigen-negative cell line. As shown in **Figures 4A–C**, sera from DT-MG3+αGalCer-immunized mice exhibited no significant binding to B16-F10 cells, whereas strong binding to EAC cells was consistently observed. This result indicated that the antibodies induced by the DT-MG3+αGalCer vaccine specifically recognized the MG7-Ag-derived epitope expressed on EAC cells, rather than mediating non-specific binding. Furthermore, mouse splenocytes were isolated after the third immunization and co-cultured with EAC cancer cells (**Figure 5A**). Results showed that splenocytes from the DT-MG3+αGalCer co-immunization group exhibited markedly higher killing rates against EAC cells than other experimental groups. Splenocytes from the DT-MG3 group displayed moderate cytotoxicity, whereas those from the MG3+αGalCer group showed cytotoxicity comparable to the PBS group. These results indicated that DT-derived T helper epitopes and αGalCer synergistically enhanced splenocyte cytotoxicity to eliminate tumor cells. It should be noted that the whole-splenocyte cytotoxicity assay may include contributions from NK cells and iNKT cells in addition to CD8+ T cells, which likely reflects the combined effects of multiple cytotoxic cell populations.

**Figure 4:**
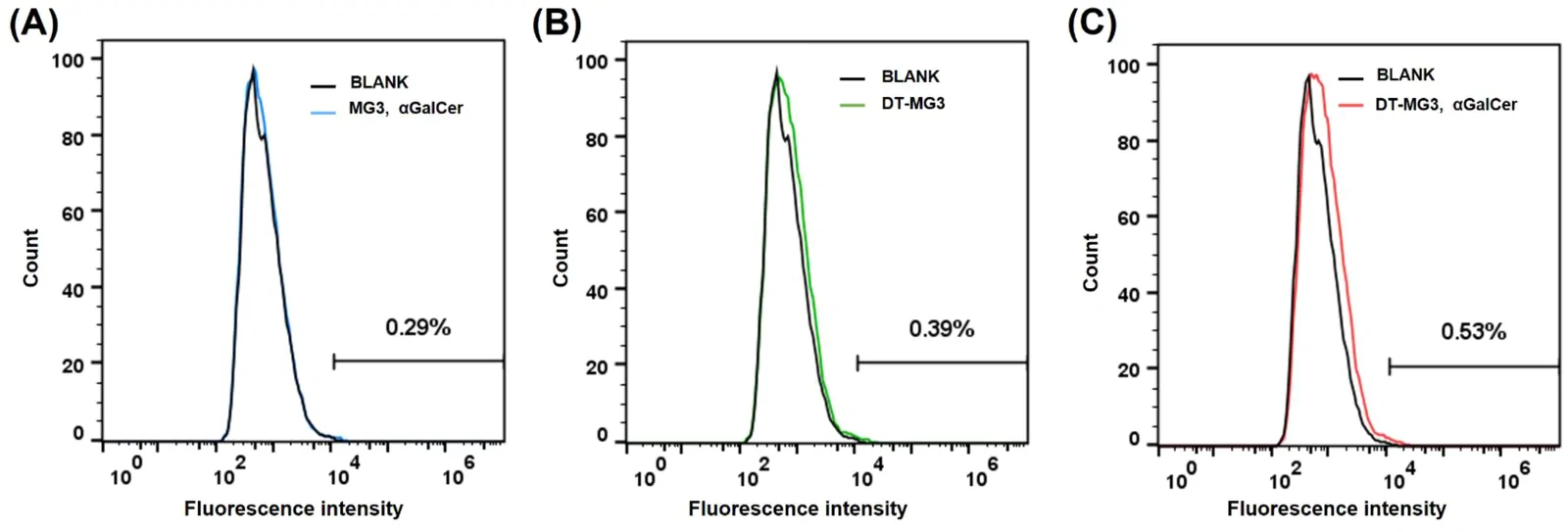
Flow cytometry analysis of serum antibody binding to B16-F10 cells: **(A)** MG3 + aGalCer group; **(B)** DT-MG3 group; **(C)** DT-MG3 + aGalCer group.

**Figure 5:**
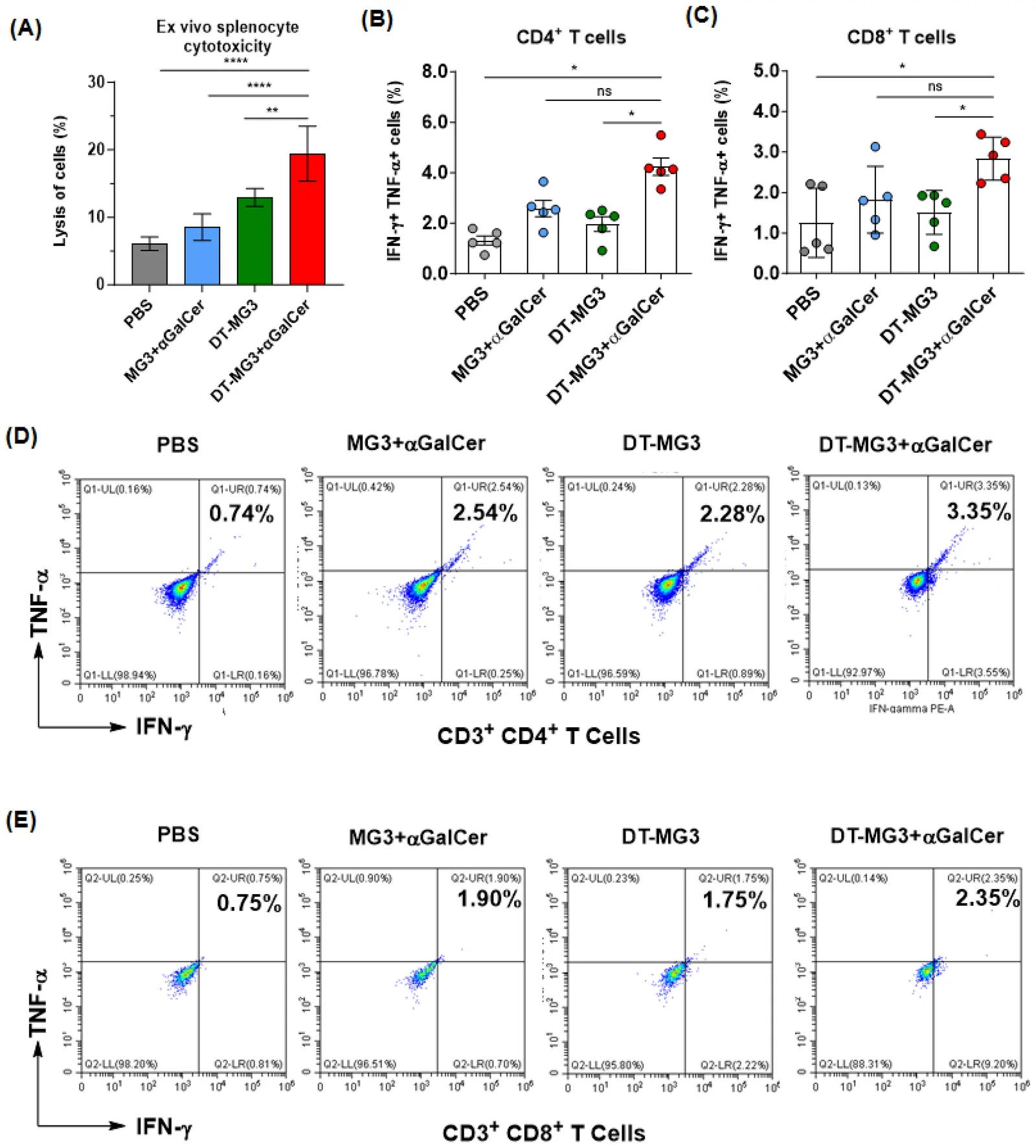
Evaluation of cellular immune responses. **(A)** Ex vivo splenocyte cytotoxicity assay. **(B, C)** Percentages of CD4^+^ and CD8^+^ splenocytes secreting IFN-γ and TNF-α. **(D, E)** Representative flow cytometry plots. Data are presented as mean ± SEM, statistical significance was analyzed by one-way ANOVA with Tukey’s multiple comparisons test: *P < 0.05, **P < 0.01, ****P < 0.0001, and ns indicates no significant difference.

The consistently superior performance of the full combination across multiple readouts (IgG titers, CDC, and ex vivo splenocyte cytotoxicity assay) provides strong evidence for a synergistic enhancement. Mechanistically, covalent DT-MG3 conjugation may facilitate linked help through presentation by the same APC, while αGalCer activates NKT cells to promote DC maturation and cross-presentation. These two pathways are complementary and together enhance the immunogenicity of the MG3 antigen.

### 3.3 DT-MG3+αGalCer vaccine enhanced antigen-specific cellular immunity

Cellular immunity plays a critical role in anti-tumor immune responses by promoting the activation of IFN-γ+ and TNF-α+ T cells, which inhibit tumor growth and eliminate tumor cells. To further explore the antigen-specific cellular immune responses elicited by the vaccine, intracellular cytokine staining (ICS) was performed on splenocytes isolated at day 14 following the final immunization (**Figures 5B–E**). The highest proportion of IFN-γ+ TNF-α+ double-positive CD4^+^ T cells (4.24%) was induced by the DT-MG3+αGalCer group, which was higher than that induced by the DT-MG3 group (1.97%), demonstrating the importance of αGalCer adjuvant in enhancing antigen-specific immune responses. A similar trend was observed in CD8+ T cells, with the DT-MG3+αGalCer group induced the highest percentage of IFN-γ+ TNF-α+ double-positive cells. These findings indicated that diphtheria toxin-derived T cell epitopes and the NKT cell agonist αGalCer synergistically enhanced antigen-specific cellular immune responses.

### 3.4 DT-MG3+αGalCer vaccine induced potent antitumor immunity

Malignant ascites is a common complication of advanced gastric cancer associated with extremely poor prognosis (43). Widespread dissemination of tumor cells within the peritoneal cavity, accompanied by massive malignant ascites accumulation, represents a major bottleneck limiting the efficacy of clinical treatment. The Ehrlich ascites carcinoma (EAC) model can closely recapitulate the pathological features of peritoneal metastasis and malignant ascites formation in advanced gastric cancer, reliably reflecting tumor proliferation, invasion and ascites accumulation in the peritoneal microenvironment, which is highly consistent with the clinical course of advanced gastric cancer (44). The EAC model has been extensively validated in previous MG7-Ag vaccine studies as a reliable platform for evaluating antitumour immunity, effectively recapitulating the peritoneal dissemination and malignant ascites accumulation characteristic of advanced gastric cancer. As shown in **Figures 6A–D**, the DT-MG3+αGalCer vaccine substantially prolonged the survival of ascites tumor-bearing mice, and both body weight loss and ascites accumulation were markedly alleviated, indicating that this candidate vaccine effectively suppressed ascites tumor progression. In contrast, treatment with DT-MG3 alone or MG3+αGalCer only exerted moderate antitumor effects, which were markedly weaker than those in the DT-MG3+αGalCer vaccine. Collectively, these results demonstrated that diphtheria toxin-derived Th epitopes and the NKT cell agonist αGalCer could synergistically enhance antitumor immune responses *in vivo*.

**Figure 6:**
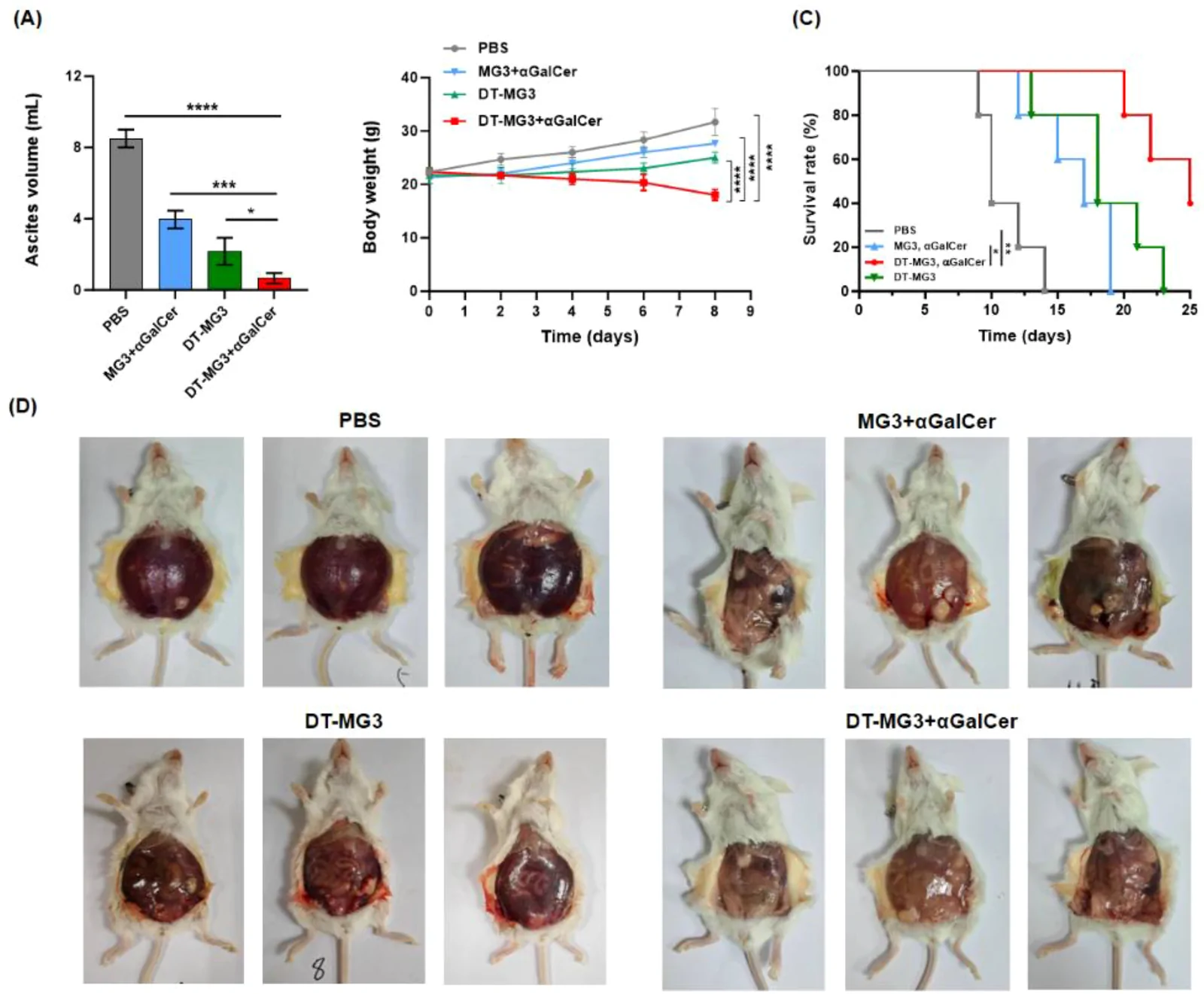
**(A)** Ascites volume in immunized mice, **(B)** Body weight growth curves of immunized mice, Data are presented as mean ± SEM, statistical significance was analyzed by one-way ANOVA with Tukey’s multiple comparisons test: *P < 0.05, ***P < 0.001, ****P < 0.0001, and ns indicates no significant difference. **(C)** survival rates of mice in additional survival experiments, asterisks show significant difference based on the log-rank test: *P < 0.05, **P < 0.01. **(D)** Representative images of ascites tumors.

### 3.5 The biosafety assessment of candidate vaccines

To evaluate the biosafety of the vaccine candidate, serum hepatic and renal function parameters were measured on day 42 after completion of the immunization regimen to assess the recovery of liver and kidney function in mice following the immunization course. Alanine aminotransferase (ALT) was used to assess hepatocyte injury, while urea (UREA) and creatinine (CREA) were used to evaluate renal detoxification function (**Figures 7A, B**). Results showed only slight differences in values among all vaccine groups, with no significant abnormalities in liver or kidney function observed compared to the PBS group. Additionally, mice were euthanized on day 42, and major organs including the heart, liver, spleen, lungs, kidneys, and brain were collected for histopathological examination. The harvested tissues were fixed, embedded, and sectioned for observation. No obvious inflammatory infiltration or other pathological changes were found in the main organs of mice treated with the candidate vaccine, providing preliminary evidence for the safety profile of the vaccine.

**Figure 7:**
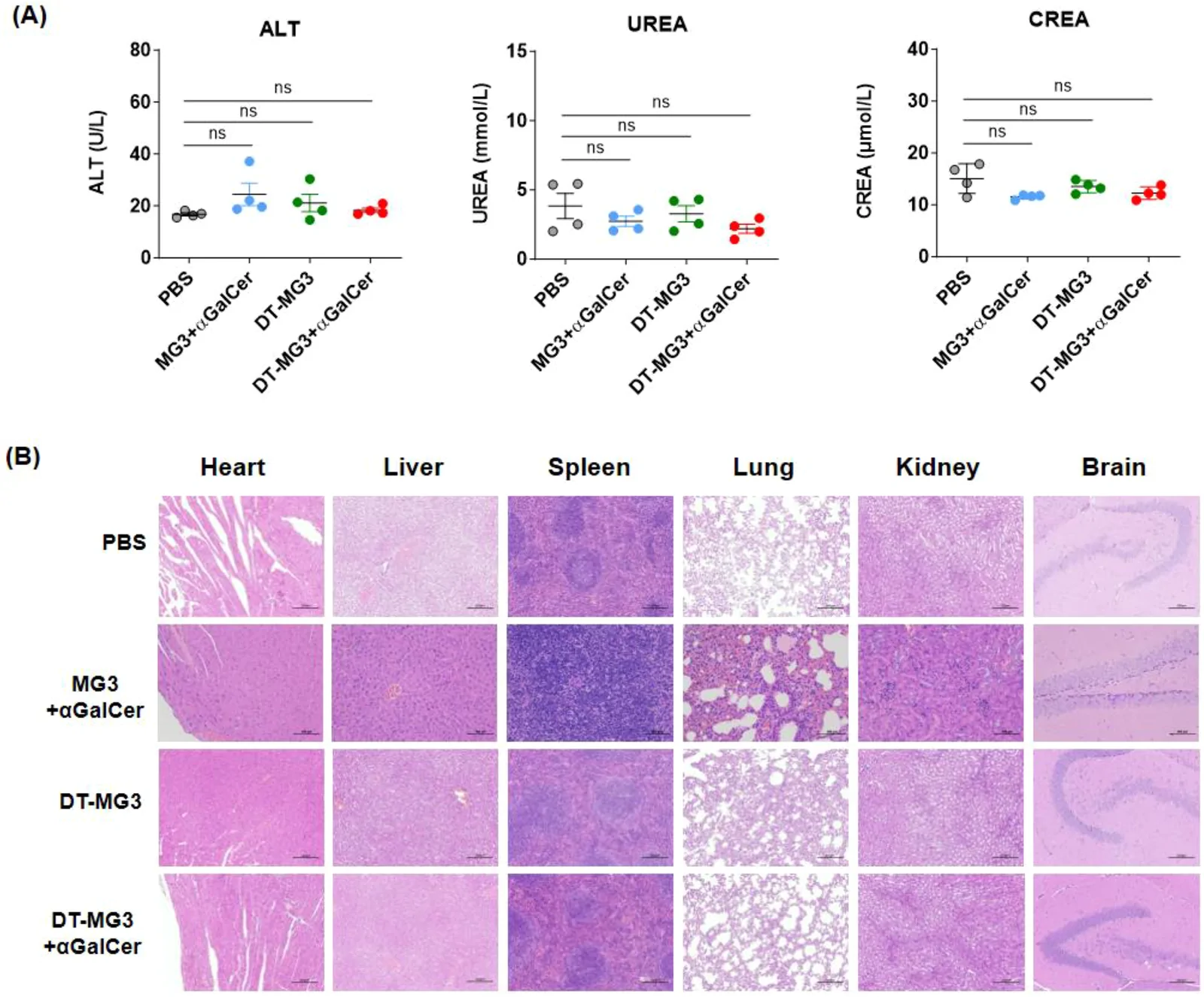
**(A)** Analysis of blood chemical markers in immunized mice on day 42 (n=4). **(B)** Representative results of H&E staining for major organs of vaccinated mice: heart, liver, spleen, lung, kidney, and brain on day 42 (n=3). Scale bar = 100 μm. Data are presented as mean ± SEM, statistical significance was analyzed by one-way ANOVA with Tukey’s multiple comparisons test: ns indicates no significant difference.

## 4 Discussion

The clinical management of advanced gastric cancer remains challenging, which was predominantly characterized by extensive local immunosuppression and dismal prognosis. Although therapeutic cancer vaccines have been recognized as a promising strategy to restore systemic antitumor immunity, clinical application of such vaccines was greatly restricted by the weak intrinsic immunogenicity of the tumor-associated antigen MG1, which failed to elicit sufficient immune responses. In the present study, a novel liposomal vaccine against gastric cancer was constructed and systematically characterized. This multicomponent vaccine was formulated with a trivalent tumor antigen MG3, a T helper epitope derived from diphtheria toxin (DT), and the NKT cell agonist αGalCer. These results demonstrated that robust and broad-spectrum antitumor immune responses were elicited by this vaccine platform, and tumor progression was markedly suppressed with prolonged survival observed in a highly aggressive murine ascites tumor model.

The superior immune potency obtained from DT-MG3 combined with αGalCer is presumably derived from synergistic cooperation between adaptive helper T-cell priming and innate NKT activation (45). The conjugated DT helper epitope may supply sufficient MHC-II-dependent costimulatory signals to CD4^+^ T cells, which in turn facilitates efficient B-cell differentiation and antibody isotype switching. Meanwhile, αGalCer-triggered NKT cell activation likely accelerates dendritic cell maturation and antigen cross-presentation, bridging innate and adaptive immune cascades cooperatively.

Monovalent MG1 peptide antigens were rapidly degraded *in vivo*, and efficient cross-linking of B-cell receptors was not accomplished, which frequently led to the induction of immune tolerance. To overcome this limitation, molecular modification was implemented in vaccine design, and a trivalent MG3 antigen was generated by tandem repetition of MG1 sequences. Sufficient B-cell activation was enabled by the multivalent property of MG3 antigens. Nevertheless, durable immune protection could not be established by mere B-cell receptor cross-linking in the absence of secondary costimulatory signals. Accordingly, the DT_331–345_ epitope derived from diphtheria toxin was covalently conjugated to antigen fragments, and MHC-II-restricted CD4+ T-cell help was thereby supplied. Trivalent MG3 design may overcome the poor immunogenicity limitation of monomeric MG1 peptides. Multimeric antigen structure presumably enhances B-cell receptor cross-linking and reduces rapid peptide degradation *in vivo*. Compared with monovalent constructs, multivalent antigens are likely to initiate stronger initial B-cell clustering, while covalent linkage with DT epitope further complements indispensable CD4^+^ helper signals for durable humoral responses.

As an essential mediator linking innate and adaptive immunity, αGalCer was presented by CD1d molecules on antigen-presenting cells, after which NKT cells were rapidly activated and massive production of immunoregulatory cytokines was stimulated (46, 47). Early innate immune cytokines, including interleukin-4 and interferon-γ, were abundantly secreted by formulations containing αGalCer. Notably, prominent synergistic effects were achieved by the combination of the DT epitope and αGalCer. Potent antibody class switching was driven by the liposomal DT-MG3+αGalCer vaccine, as evidenced by drastically elevated IgG levels and maintained low IgM production. In addition to the robust induction of IgG1, the expression of Th1-associated antibody subclasses (IgG2a, IgG2b and IgG3) was also markedly upregulated, which conferred critical clinical relevance to this vaccine candidate. Cell-based assays demonstrated that stronger binding avidity toward EAC tumor cells was exhibited by the serum collected from the DT-MG3+αGalCer group, and efficient complement-dependent tumor cell lysis was mediated. Superior therapeutic potency was verified in this combination group when compared with the monovalent antigen group and adjuvant-free control group.

Aside from humoral immunity, the effective eradication of solid tumors was highly dependent on multifunctional cellular immune responses. Intracellular cytokine staining assays indicated that the highest frequencies of IFN-γ+ TNF-α+ double-positive CD4^+^ and CD8+ T cells were induced by the DT-MG3+αGalCer vaccine. Tumor cell apoptosis was directly triggered by TNF-α, while the expression of MHC-I molecules on tumor cells was upregulated by IFN-γ to enhance the susceptibility of tumor cells to immune recognition (48). Potent cytotoxicity against EAC cells was validated in splenocytes isolated from mice receiving combined vaccination in cytotoxic T lymphocyte killing assays. Previous studies have identified the MG1 peptide (KPHVHTK) mainly as a B-cell epitope derived from the MG7-Ag sequence. Notably, our ELISA and ICS results suggest that, in addition to serving as a B-cell epitope, MG1 may also function as a potential T-cell epitope capable of inducing T-cell immune responses against tumour cells.

Furthermore, the therapeutic potential of our formulation was verified in the EAC ascites tumor model, a classic preclinical platform mimicking peritoneal metastasis of advanced gastric cancer. Marked suppression of malignant ascites and prolonged animal survival in the combinatorial vaccine group likely rely on joint functions of cellular killing and complement-dependent cytotoxicity. Single-component formulations fail to achieve satisfactory *in vivo* protection, presumably because isolated DT epitope or αGalCer alone cannot simultaneously activate both innate and adaptive immune compartments. In addition, unchanged liver and kidney biochemical parameters and intact organ histopathological morphology imply that the optimized liposomal delivery system may guarantee great *in vivo* biosafety at the applied immunization dose, potentially reducing the risk of systemic autoimmune injury for subsequent translational research.

While the EAC model provides valuable proof-of-concept evidence for the antitumour efficacy of the DT-MG3+αGalCer vaccine, further studies in gastric cancer-specific syngeneic or genetically engineered mouse models are warranted to confirm its translational potential. In addition, how this vaccine reshapes the suppressive local tumor microenvironment remains unclarified in the present study. Future investigations may combine this peptide vaccine with immune checkpoint blockades, which could potentially further sustain long-term antitumor immune memory; additional preclinical studies using advanced animal models are warranted to validate its clinical applicability.

## 5 Conclusions

To improve the immunogenicity of the gastric cancer-associated antigen MG1, a novel liposomal anti-gastric cancer vaccine incorporated with trivalent MG3 antigen, diphtheria toxin-derived Th epitope and αGalCer was successfully constructed in this study. Sufficient CD4^+^ T cell help was provided by covalently conjugated DT epitope, and the production of antigen-specific IgG was significantly enhanced. The crosstalk between innate and adaptive immunity was further regulated by αGalCer, thereby facilitating antibody subtype switching and strengthening complement-dependent tumor killing. Moreover, potent cellular immune responses were triggered by the combined vaccine, and the frequencies of functional IFN-γ+ TNF-α+ double-positive T cells were highly elevated to exert robust tumoricidal effects. *In vivo* evaluation based on the EAC malignant ascites model demonstrated that ascites accumulation was markedly suppressed, and the survival of tumor-bearing mice was prominently prolonged without obvious systemic toxicity. Collectively, this liposomal vaccine platform represents a promising candidate for clinical immunotherapeutic application against advanced gastric cancer.

## Data Availability

The original contributions presented in the study are included in the article/**Supplementary Material**. Further inquiries can be directed to the corresponding authors.

## Supplementary material

Scheme 1 Preparation of the peptide antigens: **(A)** DT-MG1; **(B)** DT-MG3.
