# Supplementary material for: A trivalent gastric cancer antigen conjugated to a diphtheria toxin epitope synergizes with an NKT cell agonist to boost antitumor immunity
Source: Front Immunol. 2026 Sep 24;17:1903458. doi: 10.3389/fimmu.2026.1903458 (PMC13645768; doi:10.3389/fimmu.2026.1903458)
Supplement: Supplementary file 1 [file Table1.docx]

*Supporting Information*

**A trivalent gastric cancer antigen conjugated to a diphtheria toxin epitope synergizes with an NKT cell agonist to boost antitumor immunity**

Jing-Jing Du^1,†^, Rong-Ling Liu^1,†^, Yao-Wei Bai^2,†^, Jin-Xuan Su^2^, Yuan-Mei Zheng^1^, Zhen-Hong Su^1^, Shi-Hao Zhou^1^, Jun Guo^3^, Dong-Yuan Wang^2,*^, and Ru-Yan Zhang^1,*^

^1^Hubei Provincial Key Laboratory of Occurrence and Intervention of Kidney Diseases, Hubei Provincial Engineering Research Center of Immunotherapy Drugs for Renal Tumors, Hubei Polytechnic University, College of Medicine, Huangshi 435003, China

^2^Department of Pharmacy, Union Hospital, Tongji Medical College, Huazhong University of Science and Technology, Wuhan 430022, China

^3^National Key Laboratory of Green Pesticide, International Joint Research Center for Intelligent Biosensing Technology and Health, College of Chemistry, Central China Normal University, Wuhan 430079, China

^†^These authors contributed equally to this work.

*Corresponding Author

E-mail: [wangdy2019@hust.edu.cn,](mailto:wangdy2019@hust.edu.cn,) ryzhang23@163.com

**Table of Contents**

[1. General methods	3](#_Toc239017425)

[2. Experimental procedures for the synthesis of 1-5	4](#_Toc239017426)

[2.1 General synthesis of MG1 (1)	4](#_Toc239017427)

[2.2 General synthesis of MG3 (2)	5](#_Toc239017428)

[2.3 General synthesis of DT-MG1 (3)	6](#_Toc239017429)

[2.4 General synthesis of DT-MG3 (4)	8](#_Toc239017430)

[2.5 General synthesis of D-Biotin-MG1 (5)	9](#_Toc239017431)

[3. Characteristics and Immune Assessment	10](#_Toc239017432)

[3.1 Vaccine formulation	10](#_Toc239017433)

[3.2 Immunization of mice	10](#_Toc239017434)

[3.3 Stability characterization of liposomal formulations	11](#_Toc239017435)

[3.4 Assessment of IgG responses	12](#_Toc239017436)

[3.5 CDC assay containing control groups.	12](#_Toc239017437)

[3.6 Preliminary evaluation of the safety of weight change	13](#_Toc239017438)

[4. Analytical data of compounds 1-5	13](#_Toc239017439)

# 1. General methods

All Fmoc-protected amino acids were purchased from GL Biochem (Shanghai, China). N,N-Dimethylformamide (DMF) was stored with 4 Å molecular sieves for drying. Unless otherwise specified, all other reagents and solvents were used without further purification. DMEM, RPMI-1640 and fetal bovine serum (FBS) applied in cell culture were acquired from Gibco. Distearoylphosphatidylcholine (DSPC) was supplied by Tokyo Chemical Industry (TCI), and cholesterol was obtained from Energy Chemical (Shanghai, China). Horseradish peroxidase-conjugated goat anti-mouse IgG and IgM antibodies were provided by Jackson ImmunoResearch Labs; horseradish peroxidase-conjugated goat anti-mouse IgG1, IgG2a, IgG2b and IgG3 antibodies were purchased from SouthernBiotech. HPLC analysis was conducted on an Agilent 1260 system equipped with semi-preparative C18/C8 reversed-phase columns (Agilent, 250 × 9.4 mm, 5 µm) at a flow rate of 4.0 mL/min. The collected target eluted fractions were treated by lyophilization, and high-purity peptide products were acquired.

# 2. Experimental procedures for the synthesis of 1-5

# 2.1 General synthesis of MG1 (1)

**MG1 (1)** was synthesized via Fmoc-based solid-phase peptide synthesis (SPPS) using Rink amide AM resin (77 mg, 0.05 mmol, loading capacity: 0.65 mmol/g). Standard Fmoc-protected amino acids (Fmoc-AA-OH) were coupled with the assistance of PyBOP (78 mg, 0.15 mmol, 3 eq) and DIPEA (53 μL, 0.3 mmol, 6 eq). Terminal Fmoc deprotection was performed using a 20% (v/v) piperidine solution in DMF, followed by sequential washing with DMF and DCM. Iterative amino acid coupling steps were completed in accordance with standard Fmoc-SPPS procedures. Upon the completion of full-sequence assembly, the N-terminal Fmoc protecting group was fully cleaved. The resin-bound peptide was then treated with a cleavage cocktail consisting of trifluoroacetic acid/triisopropylsilane/water (95:2.5:2.5, v/v/v) and incubated with continuous shaking for 2 h. The resulting supernatant was collected, and the residual resin was washed three times with trifluoroacetic acid. Combined filtrates were concentrated under reduced pressure, and the crude residue was lyophilized. The obtained crude product was redissolved in methanol (5 mL) and purified by semi-preparative high-performance liquid chromatography (HPLC). The compound **1** was finally characterized and detected by analytical HPLC (column: Agilent C18, gradient: water/acetonitrile + 0.1% TFA, 0 min (95:5) → 25 min (70:30) → 25.1 min (0:100) → 30 min (0:100), 30 min. R_t_ = 10.898 min. ESI-MS: Calcd for C_38_H_64_N_14_O_8_ [M+H]^+^ m/z = 845.5, [M+2H]^2+^ m/z = 423.3; observed: 845.5 [M+H]^+^, 423.4 [M+2H]^2+^.

# 2.2 General synthesis of MG3 (2)

**MG3 (2)** was synthesized via Fmoc-SPPS using resin-bound peptide S1. The coupling of standard Fmoc-protected amino acids (Fmoc-AA-OH) was performed with PyBOP (78 mg, 0.15 mmol, 3 eq) and DIPEA (53 μL, 0.3 mmol, 6 eq). After the completion of coupling for all building blocks, a cleavage mixture consisting of trifluoroacetic acid/triisopropylsilane/water (95:2.5:2.5, v/v/v) was added to the reactor, and the reaction was shaken for 2 h at a constant temperature. The filtrate was collected, and the resin was washed three times with trifluoroacetic acid. The solvent was removed under vacuum, and the resulting residue was lyophilized. The crude product was dissolved in 5 mL of methanol and further purified by semi-preparative HPLC. The compound **2** was finally characterized and detected by analytical HPLC (column: Agilent C8, gradient: water/acetonitrile + 0.1% TFA, 0 min (94:6) → 25 min (69:31) → 25.1 min (0:100)→ 30 min (0:100), 30 min. R_t_ = 8.806 min; ESI-MS: Calcd for C_102_H_162_N_36_O_22_ [M+2H]^2+^ m/z =1123.1, [M+3H]^3+^ m/z =749.1; observed: 1123.2 [M+2H]^2+^, 749.2 [M+3H]^3+^.

# 2.3 General synthesis of DT-MG1 (3)

**DT-MG1 (3)** was prepared via Fmoc SPPS starting from resin-bound peptide **S1**. The coupling of conventional Fmoc-protected amino acids (Fmoc-AA-OH) was accomplished using PyBOP (78 mg, 0.15 mmol, 3 eq) and DIPEA (53 μL, 0.3 mmol, 6 eq). After the coupling of all building blocks was completed, a mixed cleavage solution of trifluoroacetic acid/triisopropylsilane/water (95:2.5:2.5, v/v/v) was added into the reactor, and the mixture was shaken for 2 h. The filtrate was collected, and the resin was washed three times with trifluoroacetic acid. The solvent was removed under reduced pressure, and the residue was lyophilized. The crude product was dissolved in 5 mL of methanol and purified by semi-preparative HPLC. Compound **3** was finally characterized and detected by analytical HPLC (column: Agilent C8, gradient: water/acetonitrile + 0.1% TFA, 0 min (80:20) → 25 min (55:45) → 25.1 min (0:100)→ 30 min (0:100), 30 min. R_t_ = 10.243 min; ESI-MS: Calcd for C_105_H_179_N_31_O_28_S [M+4H]^4+^ m/z = 589.8, [M+5H]^5+^ m/z = 472.1; observed: 589.8 [M+4H]^4+,^ 472.1 [M+5H]^5+^.

# 2.4 General synthesis of DT-MG3 (4)

**DT-MG3 (4)** was synthesized using the Fmoc-SPPS strategy, starting from the resin-bound peptide **S2**, conventional amino acids (Fmoc-AA-OH) were introduced by the addition of PyBOP (78 mg, 0.15 mmol, 3 eq) and DIPEA (53 μL 0.3 mmol, 6 eq). After coupling of all building blocks,a mixture of trifluoroacetic acid/triisopropylsilane/water (95:2.5:2.5, v/v/v) was added to the reactor and shaken for two hours. Filtrated the solution and washed the resin three times using trifluoroacetic acid, the solvent was removed in vacuo, and the residue was lyophilized. The crude product was dissolved in MeOH (5 mL) and purified by semi-preparative HPLC. The compound **4** was finally characterized and detected by analytical HPLC (column: Agilent C8, gradient: water/acetonitrile + 0.1% TFA, 0 min (85:15) → 25 min (60:40) → 25.1 min (0:100)→ 30 min (0:100), 30 min. R_t_ = 14.107 min; ESI-MS: Calcd for C_169_H_277_N_53_O_42_S [M+3H]^3+^ m/z = 1252.4, [M+4H]^4+^ m/z = 939.5; observed: 1252.4 [M+3H]^3+^, 939.5 [M+4H]^4+^.

# 2.5 General synthesis of D-Biotin-MG1 (5)

**D-Biotin-MG1 (5)** was synthesized using the Fmoc-SPPS strategy, starting from the resin-bound peptide **S1**, conventional amino acids (Fmoc-AA-OH) were introduced by the addition of PyBOP (78 mg, 0.15 mmol, 3 eq) and DIPEA (53 μL 0.3 mmol, 6 eq). After coupling of all building blocks,a mixture of trifluoroacetic acid/triisopropylsilane/water (95:2.5:2.5, v/v/v) was added to the reactor and shaken for two hours. Filtrated the solution and washed the resin three times using trifluoroacetic acid, the solvent was removed in vacuo, and the residue was lyophilized. The crude product was dissolved in MeOH (5 mL) and purified by semi-preparative HPLC. The compound **5** was finally characterized and detected by analytical HPLC (column: Agilent C8, gradient: water/acetonitrile + 0.1% TFA, 0 min (92:8) → 25 min (67:33) → 25.1 min (0:100)→ 30 min (0:100), 30 min. R_t_ = 9.458 min; ESI-MS: Calcd for C_50_H_81_N_17_O_11_S [M+2H]^2+^ m/z = 564.8, [M+H]^+^ m/z = 1128.6; observed: 564.9 [M+2H]^2+^, 1128.5 [M+H]^+^.

# 3. Characteristics and Immune Assessment

## 3.1 Vaccine formulation

The mixture of antigens **1**-**4** (4.6 nmol), adjuvants (2.3 nmol), 1,2-distearoyl-sn-glycero-3-phosphocholine (18.2 μg, 23 nmol), and cholesterol (7.1 μg, 18.4 nmol) (a 10:5:50:40 molar ratio) was dissolved in CH_2_Cl_2_ and MeOH (1:1, v/v, 2 mL) in a 4 mL round-bottomed flask. The film was then hydrated with 1.2 mL of HEPES buffer (20 mM, pH 7.5), and the resulting suspension was subjected to sonication for 15 min in an ultrasonic cleaner (100 W, 40 kHz) to enhance uniformity.

Table S1. The composition of each vaccine candidate.

| Vaccines | Antigen  (4.6 nmol) | Adjuvant  (2.3 nmol) | DSPC  (23 nmol) | Cholesterol  (18.4 nmol) |
| --- | --- | --- | --- | --- |
| A | MG1 (3.9 µg) | αGalCer (1.97 µg) | 18.20 µg | 7.10 µg |
| B | DT-MG1 (10.8 µg) | αGalCer (1.97 µg) | 18.20 µg | 7.10 µg |
| C | DT-MG1 (10.8 µg) | / | 18.20 µg | 7.10 µg |
| D | MG3 (10.3 µg) | αGalCer (1.97 µg) | 18.20 µg | 7.10 µg |
| E | DT-MG3 (17.3 µg) | αGalCer (1.97 µg) | 18.20 µg | 7.10 µg |
| F | DT-MG3 (17.3 µg) | / | 18.20 µg | 7.10 µg |

*^a^*The amounts of each component in the table are used for one injection per mouse.

## 3.2 Immunization of mice

Female BALB/c mice (4-6 weeks old) (laboratory animal license No.: SCXK(E) 2025-0018) were randomly divided into experimental and control groups. Mice (n = 5 per group) were immunized intraperitoneally with vaccines A-F on days 1, 15, and 29. Serum samples were collected on days 0, 14, 28, and 42 after the first immunization. On day 42 post-immunization, mice were euthanized and spleens were aseptically harvested. For ascites tumor model experiments, mice were acclimatized for 1 week and randomly divided into 4 groups (n = 5-7 per group). Tumor models were established by intraperitoneal injection of 100 µL logarithmic-phase EAC ascites cells resuspended in PBS. From day 8 post-modeling, treatments were administered intraperitoneally every other day for a total of 4 injections. All animal experiments were conducted in strict compliance with laboratory animal welfare and ethical guidelines.

## 3.3 Stability characterization of liposomal formulations

**Figure S1.** Stability characterization of liposomal formulations. (A-B) Particle size and PDI of three independent batches measured; (C-D) Changes in particle size and PDI over time (1, 7, 14 days). Data are presented as mean ± SEM (n=3).

## 3.4 Assessment of IgG responses

**Figure S2.** Anti-MG1 IgG response in female BALB/c mice at days 14, 28, and 42 post-vaccination with the candidate vaccine. The data are expressed as the mean ± SEM, and asterisks denote significant differences determined by one-way ANOVA with Tukey's multiple comparisons test, ** P < 0.01, * P < 0.05, ns: not significant.

## 3.5 CDC assay containing control groups.

**Figure S3.** CDC assay containing RC-inactivated, antibody alone, preimmune serum, and rabbit complement-only (RC) control groups. The data are expressed as the mean ± SEM, and asterisks denote significant differences determined by one-way ANOVA with Tukey's multiple comparisons test, **** P < 0.0001, * P < 0.05.

## 3.6 Preliminary evaluation of the safety of weight change

**Figure S4.** Assessment of the safety of vaccines by monitoring the weight changes of immunized mice.

# 4. Analytical data of compounds 1-5

**Figure S5.** HPLC chromatogram of compound **1**.

**Figure S6.** ESI-MS spectra of compound **1**.

**Figure S7.** HPLC chromatogram of compound **2**.

**Figure S8.** ESI-MS spectra of compound **2**.

**Figure S9.** HPLC chromatogram of compound **3**.

**Figure S10.** ESI-MS spectra of compound **3**.

**Figure S11.** HPLC chromatogram of compound **4**.

**Figure S12.** ESI-MS spectra of compound **4**.

**Figure S13.** HPLC chromatogram of compound **5**.

**Figure S14.** ESI-MS spectra of compound **5**.
